# Hidden variables, free choice, context-independence and all that

**DOI:** 10.1098/rsta.2023.0015

**Published:** 2024-03-18

**Authors:** Ehtibar N. Dzhafarov

**Affiliations:** Department of Psychological Sciences, Purdue University, West Lafayette, IN, USA

**Keywords:** contextuality, context-independent mapping, context-irrelevance, coupling, free choice, hidden-variable

## Abstract

This paper provides a systematic account of the hidden variable models (HVMs) formulated to describe systems of random variables with mutually exclusive contexts. Any such system can be described either by a model with free choice but generally context-dependent mapping of the hidden variables into observable ones, or by a model with context-independent mapping but generally compromised free choice. These two types of HVMs are equivalent, one can always be translated into another. They are also unfalsifiable, applicable to all possible systems. These facts, the equivalence and unfalsifiability, imply that freedom of choice and context-independent mapping are no assumptions at all, and they tell us nothing about freedom of choice or physical influences exerted by contexts as these notions would be understood in science and philosophy. The conjunction of these two notions, however, defines a falsifiable HVM that describes non-contextuality when applied to systems with no disturbance or to consistifications of arbitrary systems. This HVM is most adequately captured by the term ‘context-irrelevance’, meaning that no distribution in the model changes with context.

This article is part of the theme issue ‘Quantum contextuality, causality and freedom of choice’.

## 1. Introduction

Hidden variable models (HVMs) are arguably the main reason why contextuality and its non-locality version have acquired prominence in the foundations of quantum mechanics (QM). Ever since it was accepted that results of a measurement, such as that of a spin, are almost always random variables (with the exception of repeated sharp measurements), physicists have been interested in the possibility of ‘explaining’ such random variables as deterministic functions of some underlying sources of variability, even if as yet unknown to us, ‘hidden’. This possibility is often presented as a belief famously held by Albert Einstein, and then famously ruled out by Bell’s and Kochen–Specker’s theorems juxtaposed with QM predictions [1,2].

However, even before any detailed analysis, there is a good reason to doubt that HVMs can play an explanatory role. The reason is that the existence of a random variable of which several jointly distributed random variables are deterministic functions is ensured trivially: the properties of being jointly distributed and being functions of a single random variable are one and the same property. Conversely, variables that are not jointly distributed, as they are predicated on mutually exclusive conditions, cannot be functions of a single random variable. This means that one must have as many hidden variables as there are mutually exclusive contexts, even if they all have the same distribution. This is not to say that HVMs cannot be meaningfully constructed and interpreted. This only means that one should be careful not to attach deep physical or otherwise substantive connotations to purely mathematical and universally satisfiable representations. This is a point elaborated throughout the paper.

Here, I will synthesise some of my recent published work to provide a comprehensive and rigorous account of HVMs. The most restrictive HVM, one introduced by Bell and describing non-contextual systems with no disturbance, is known not to hold for many systems of random variables. When this happens, the constraints imposed on an HVM have to be relaxed, and this can be done in two ways: either by allowing for a dependence of the measurement outcome distributions on contexts or by allowing for an interdependence between the hidden variables and the choices of settings for the measurements. In [3], I proved the equivalence of these two options. In this paper, I present an improved and more rigorous proof. I will argue that such assumptions as freedom of choice and context-independent mapping (of hidden variables into observable ones) are merely metaphorical depictions of some basic representations of jointly distributed random variables. Next, I discuss the problem of separating disturbance (or signalling) from contextuality in the situations in which Bell’s HVM does not hold. While this is the central issue for the Contextuality-by-Default (CbD) theory [4–7], the difference between disturbance and contextuality is not apparent in the formulations of the HVMs. However, one can effectively separate disturbance from contextuality by using the consistified systems introduced in Dzhafarov [8,9]. Any system of random variables can be reformulated as an equivalent, in a well-defined sense, system that has no disturbance (is consistently connected, in the CbD terminology). The equivalence of the HVMs with context-dependent mapping and the HVMs with violations of free choice holds for these consistified systems too, but now any such HVM indicates pure contextuality. At the conclusion of the paper, I will discuss two assumptions that one could suspect to be required for the development presented, and show that, once again, they are not assumptions at all, because they are trivially satisfied in the language of random variables.

## 2. Conceptual and terminological set-up

A *system of random variables* is a double-indexed set

2.1 $$R=\{R_{q}^{c}:c\in C,q\in Q,q≺c\},$$

where Q is a set of *contents*, C is a set of *contexts*, and q≺c means that content q is measured in context c. A content q in $R_{q}^{c}$ can be viewed as a question that the random variable $R_{q}^{c}$ answers (e.g. ‘is the spin along axis q up?’, answered ‘yes/no’) or as a choice of measurements (spin along axis q) whose outcomes (up/down) are represented by $R_{q}^{c}$. The context c in $R_{q}^{c}$ indicates conditions under which $R_{q}^{c}$ is recorded, such as the set of all other measurements made together with $R_{q}^{c}$ and the spatial and temporal relations among them. The matrix below provides an example of a system of random variables:
2.2

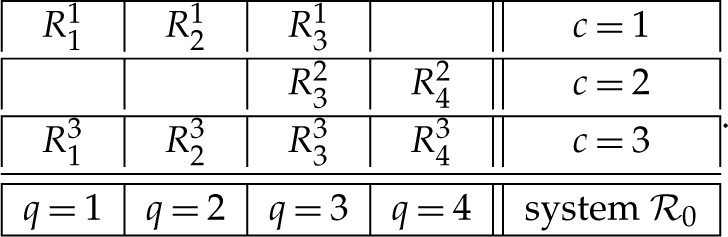

The subsystem of all random variables within a given context c is called a *bunch* (of random variables),

2.3 $$R^{c}=\{R_{q}^{c}:q\in Q^{c}\},$$

where

2.4 $$Q^{c}=\{q\in Q:q≺c\}.$$

For instance, the bunch $R^{2}$ in the system $R_{0}$ is $\left\{R_{3}^{2},R_{4}^{2}\right\}$. Any bunch $R^{c}$ is a random variable, which means that all components $R_{q}^{c}$ of $R^{c}$ are jointly distributed (are measurable functions on the same probability space). However, no two random variables from different bunches have a joint distribution, they are *stochastically unrelated* (are measurable functions on distinct probability spaces). Indeed, consider what a joint distribution of $R_{q}^{c}$ and $R_{q^{\prime }}^{c^{\prime }}$ with $c\neq c^{\prime }$ could look like (X and Y being any measurable sets):
2.5

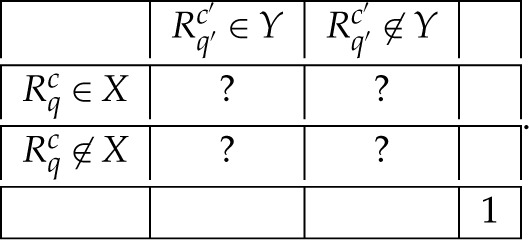

The question marks cannot be all replaced with zeros because they must sum to one. At the same time, any non-zero joint probability would indicate that $R_{q}^{c}$ and $R_{q^{\prime }}^{c^{\prime }}$ co-occur, which would contradict the fact that c and $c^{\prime }$ are mutually exclusive contexts.

## 3. Hidden variable models

### (a) (Excessively) general HVM

Let us begin with the most general possible HVM, denoted $HVM_{Gen}$:

3.1 $$R^{c}=\alpha (Q^{c},\Lambda^{c}(c),c).$$

The function α returns as its value an indexed set, and the dependence of α on $Q^{c}$ should be understood as its indexing, matching the indexing of $R^{c}$. Thus, for system $R_{0}$ in (2.2), $Q^{2}=\{3,4\}$, and the $HVM_{Gen}$ representation for $R^{2}=(R_{3}^{2},R_{4}^{2})$ is

3.2 $$\begin{array}{rl} \alpha (Q^{2},\Lambda^{2}(2),2) & \;=({Proj}_{q=3}\alpha (Q^{2},\Lambda^{2}(2),2),{Proj}_{q=4}\alpha (Q^{2},\Lambda^{2}(2),2))\\ & \;=(\alpha (3,\Lambda^{2}(2),2),\alpha (4,\Lambda^{2}(2),2)), \end{array}$$

where ${Proj}_{q}V$ stands for the q-indexed component of the indexed set V.^1^

One can present this model graphically as
3.3

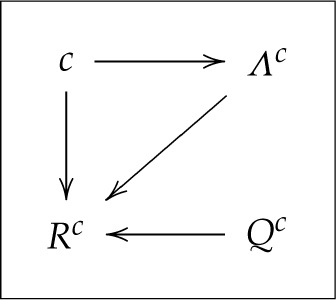

The arrows a→b in this and subsequent diagrams (where b is a random variable and a is a random variable or a parameter) should be read as ‘different values of a may result in different distributions of b.’

$HVM_{Gen}$ is not a falsifiable model, it can be applied to any system of random variables. This can be demonstrated by simply putting $\Lambda^{c}(c)=R^{c}$, with the stand-alone c in α becoming a dummy argument, and $Q^{c}$ extracted from $\Lambda^{c}(c)$ as its indexing set.

### (b) Context-independent mapping without free choice

The argument just presented shows that the direct dependence of the distribution of $R^{c}$ on c can be eliminated:

3.4 $$R^{c}=\beta (Q^{c},\Lambda^{c}(c)),$$

or graphically,
3.5

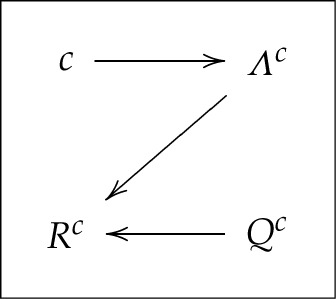

Although not obvious at first glance, this HVM would traditionally be interpreted as a model with a context-independent mapping of $\Lambda^{c}(c)$ into $R^{c}$ (no arrow from c to $R^{c}$) but with generally compromised freedom of choice (the distribution of $\Lambda^{c}$ may depend on c).

I will denote this model $HVM_{+CIM}^{-FC}$, using the self-evident abbreviations. We have established that $HVM_{Gen}$ can always be reduced to $HVM_{+CIM}^{-FC}$. Using again as an example system $R_{0}$ in (2.2), the $HVM_{+CIM}^{-FC}$ representation for $R^{2}=(R_{3}^{2},R_{4}^{2})$ is

3.6 $$\beta (Q^{2},\Lambda^{2}(2))=(\beta (3,\Lambda^{2}(2)),\beta (4,\Lambda^{2}(2))).$$

Freedom of choice in the QM literature is usually discussed in terms of the relationship between one’s choice of c and the hidden variable $\Lambda^{c}(c)$. This means that c is treated as a random variable (which is a dubious viewpoint, see [3]), and freedom of choice means that c and $\Lambda^{c}$ are stochastically independent. In my representation of HVMs, c is always a deterministic parameter, which, with respect to the traditional view, simply means that all random variables in the model are conditioned on fixed values of c. Any restriction of freedom of choice in the traditional sense then translates into a dependence of the distribution of $\Lambda^{c}$ on c. As a special case, this also applies to the possibility that c is a function of the hidden variable, $c=f(\Lambda^{c})$, which may possibly be interpreted as a depiction of superdeterminism: in an $HVM_{+CIM}^{-FC}$, one simply replaces this function with $\Lambda^{c}(c)$, defined by $f(\Lambda^{c}(c))=c$.

### (c) Free choice without context-independent mapping

It is further possible to transform $HVM_{+CIM}^{-FC}$ into a model that is, in a sense, its reverse. Given (3.4), one can form an arbitrary coupling of $\Lambda^{c}$ for all contexts c,^2^

3.7 $$\Gamma :=\{\Lambda^{c}(c):c\in C\},$$

and then create, for every c∈C, a distributional copy $\Gamma^{c}$ of Γ, so that these copies are pairwise stochastically unrelated. Then

3.8 $$\Lambda^{c}(c)={Proj}_{c}\Gamma^{c}$$

and

3.9 $$R^{c}=\beta (Q^{c},{Proj}_{c}\Gamma^{c})=\gamma (Q^{c},\Gamma^{c},c),$$

where the variables $\Gamma^{c}$ (as indicated by the lack of c as their argument) have one and the same distribution for all c∈C. Note that we cannot eliminate the index c in $\Gamma^{c}$, because $R^{c}=\gamma (Q^{c},\Gamma ,c)$ would make all $R^{c}$ jointly distributed.

The traditional interpretation of the HVM described by (3.9) would be that the freedom of choice is not compromised here, but context-independence is generally violated. Using our graphical representation,
3.10

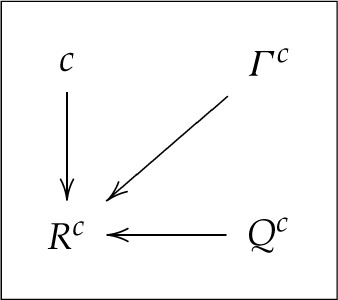


I will denote this model $HVM_{-CIM}^{+FC}$. We have established that $HVM_{+CIM}^{-FC}$ implies (can be translated into) $HVM_{-CIM}^{+FC}$. Using our example of system $R_{0}$ in (2.2), the $HVM_{-CIM}^{+FC}$ representation for $R^{2}=(R_{3}^{2},R_{4}^{2})$ is

3.11 $$\gamma (Q^{2},\Gamma^{2},2)=(\gamma (3,\Gamma^{2},2),\gamma (4,\Gamma^{2},2)).$$

### (d) Free choice with context-independent mapping

Both $HVM_{+CIM}^{-FC}$, and $HVM_{-CIM}^{+FC}$ can be viewed as deviations from their special case

3.12 $$R^{c}=\delta (Q^{c},\Gamma^{c}),$$

or, graphically,
3.13

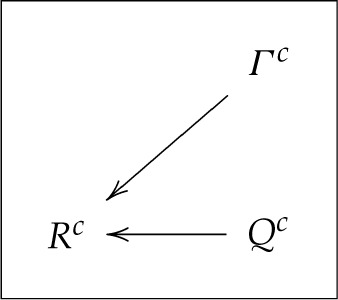

where the random variables $\Gamma^{c}$ for all c∈C are identically distributed and pairwise stochastically unrelated. This model can be denoted $HVM_{+CIM}^{+FC}$, as it satisfies both freedom of choice and context-independence in the mapping of $\Gamma^{c}$ into $R^{c}$. In our example of system $R_{0}$ in (2.2), the $HVM_{+CIM}^{+FC}$ representation for $R^{2}=(R_{3}^{2},R_{4}^{2})$ is

3.14 $$\delta (Q^{2},\Gamma^{2})=(\delta (3,\Gamma^{2}),\delta (4,\Gamma^{2})).$$

Unlike the previous two HVMs, this one is a true model, as it is falsifiable. The latter is demonstrated, for instance, by relating predictions of QM to the Bell-type [1] and Kochen–Specker-type theorems (in addition to the original references [1,2] see, e.g. [10–12]). The Bell-type theorems establish necessary and sufficient conditions for a system of random variables to be described by $HVM_{+CIM}^{+FC}$, which can then be shown to fail for some QM systems. In the Kochen–Specker-type theorems one constructs systems of random variables in accordance with QM, and then demonstrate that they cannot be described by $HVM_{+CIM}^{+FC}$.

### (e) Equivalence theorem and its consequences

Combining the implications in §§3b,c,

3.15 $$HVM_{Gen}\Rightarrow HVM_{+CIM}^{-FC}\Rightarrow HVM_{-CIM}^{+FC},$$

and observing that $HVM_{-CIM}^{+FC}$ is a special case of $HVM_{Gen}$, we obtain the following statement.

**Theorem 3.1.**

*The models* $HVM_{Gen}$, $HVM_{+CIM}^{-FC}$ *and* $HVM_{-CIM}^{+FC}$ *are pairwise equivalent*:
3.16

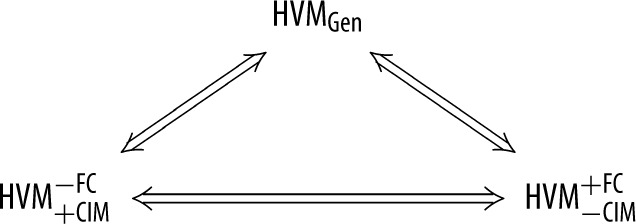

Let us consider two consequences of this theorem. One of them is that when $HVM_{+CIM}^{+FC}$ is not applicable to a system, one can arbitrarily choose between describing the system in the language of $HVM_{+CIM}^{-FC}$ or in the language of $HVM_{-CIM}^{+FC}$. In particular, one can always use one and the same measure for the degree of deviation of these two HVMs from $HVM_{+CIM}^{+FC}$:
3.17

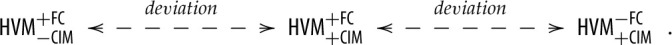

A special case of this corollary, for a particular system of random variables, is presented in [13].

The second consequence of the theorem is that $HVM_{+CIM}^{-FC}$ and $HVM_{-CIM}^{+FC}$ are both unfalsifiable, either of them can describe any system of random variables. This follows from the demonstration, at the end of §3(a), that $HVM_{Gen}$ is unfalsifiable, in fact, even in the form of $HVM_{+CIM}^{-FC}$. This, in combination with the inter-translatability of $HVM_{+CIM}^{-FC}$ and $HVM_{-CIM}^{+FC}$, should make one skeptical about interpreting the dependence of the distribution of $\Lambda^{c}$ on c in terms of freedom of choice, in any substantive meaning of these words, and interpreting an arrow from c to $R^{c}$ as a physical influence exerted by the context. Their complete equivalence and empirical emptiness (universal applicability) suggest the view that $HVM_{+CIM}^{-FC}$ and $HVM_{-CIM}^{+FC}$ are purely mathematical descriptions of the joint distributions within bunches of random variables and of the differences between them.

This view does not change if one constrains or even completely specifies all distributions and functions in the formulation of $HVM_{+CIM}^{-FC}$ or $HVM_{-CIM}^{+FC}$, making them thereby predictive and falsifiable. The inter-translatability of the two types of models holds irrespective of their falsifiability. Moreover, a completely specified HVM can always be thought of as a corresponding unconstrained HVM after it has been applied to the system predicted by the completely specified HVM. Clearly, the ontological interpretation of a model (say, $HVM_{+CIM}^{-FC}$) does not depend on whether it has been applied to a particular system of random variables, because this does not change the facts that (A) it could have been applied to any other system, and (B) it can be translated into an HVM of a completely different nature (in this case, $HVM_{-CIM}^{+FC}$).

This is not to say that the notions of freedom of choice and context-(in)dependent mapping may not be assigned substantive meanings and be propitiously used in physical or other scientific theories. One should, however, distinguish HVMs per se from scientific theories that predict specific systems of random variables and therefore HVM representations thereof. My only point here is that these substantive meanings belong to the parts of theories extraneous to the HVMs to which the theories lead. In other words, these meanings cannot be derived from the HVMs themselves, from the fact that a system can be described by $HVM_{+CIM}^{-FC}$ or $HVM_{-CIM}^{+FC}$ (or even $HVM_{Gen}$, combining the two)—because any system can, and by any of them. The language of HVMs as understood in this paper (and in most discussions of the HVMs in the foundations of physics, beginning with Bell’s work) is simply too crude to capture certain substantive notions and distinctions. We will see below that it is sometimes too crude even to depict the difference between much more clear-cut notions of contextuality and signaling. A simple analogy may help to understand this. Any real-valued random variable R can be generated by applying an appropriate transformation f to a variable U uniformly distributed between 0 and 1. As one observes values of R, it is possible that there is a computer program that de facto computes them by first generating values of U and then applying to them the function f. If this is known from some extraneous source of knowledge, then we have a valid naturalistic interpretation of the model R=f(U), which then acquires a privileged status over other representations of R (such as R=g(E), for an exponentially distributed E). However, such an interpretation cannot be derived from the fact that R is representable as f(U)—because this representation is mathematically guaranteed, and moreover, can be replaced with other representations (referring, e.g. to the same R=g(E)).

The terms freedom of choice and context-(in)dependent mapping may still be conveniently used as labels for HVM components, provided one does not impute to them their colloquial, physical, or philosophical connotations. Moreover, the conjunction of these two notions does have a substantive meaning, because $HVM_{+CIM}^{+FC}$ is a falsifiable model which de facto does not apply to some QM systems of random variables. In [3], I argued that the notions in question should only be used in conjunction: ‘one cannot accept local causality without free choice, because denying free choice is equivalent to denying local causality’ (local causality being the specific form of context-independent mapping used by Bell in the discussion published in [14]). While the present paper only strengthens this assertion, I would like to add here that one can very well decide to abandon the terms freedom of choice and context-(in)dependent mapping altogether, and use instead a simpler way to characterise $HVM_{+CIM}^{+FC}$. Namely, this is the model in which context c is irrelevant for determining any distributions involved (which includes the distribution of the hidden variable $\Lambda^{c}$ and the distribution of the observable bunch $R^{c}$). Therefore, $HVM_{+CIM}^{+FC}$ can be referred to as the model satisfying the assumption of *context-irrelevance*.

## 4. Contextuality in consistently connected systems

We have managed so far to discuss HVMs without involving the notion of (*non*)*contextuality*. It is now time to involve it. The traditional definition of this notion simply coincides with that of $HVM_{+CIM}^{+FC}$: a system of random variables is non-contextual (or, for distributed systems, local) if it is described by this HVM, and a system that cannot be so described is contextual. One consequence of this definition is that a non-contextual system must be *consistently connected*. The latter is a CbD term for what is usually called in QM *non-disturbance* or *non-signaling*: in a consistently connected system, any two random variables sharing a content, $R_{q}^{c}$ and $R_{q}^{c^{\prime }}$, have the same distribution. *Inconsistent connectedness* (disturbance, signalling) therefore makes a system contextual. This definition makes the class of contextual systems too large and heterogeneous, and CbD offers a more analytic approach, presented in the next section. For now, however, let us confine consideration to consistently connected systems.^3^

The main consequence of R being described by $HVM_{+CIM}^{+FC}$ is as follows. With reference to (3.12), construct the random variable S defined by

4.1 S=δ(Q,Γ),

where Γ has the same distribution as $\Gamma^{c}$ in (3.12). The variable S is called a *reduced coupling* of the system R [15]. Its (jointly distributed) elements are indexed by the elements of Q, and for any c∈C, we have

4.2 $$R^{c}\overset{d}{=}{Proj}_{Q^{c}}S,$$

where $\overset{d}{=}$ indicates equality of distributions. Thus, for our system $R_{0}$ in (2.2), the reduced coupling has the form $S=\{S_{1},S_{2},S_{3},S_{4}\}$, and the condition (4.2) means that in the matrix
4.3

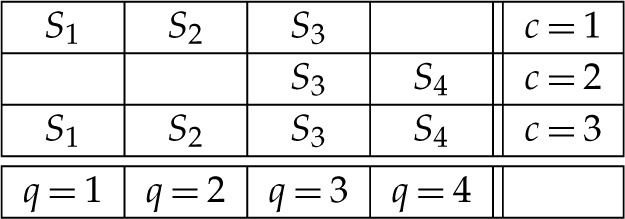

the rows are distributed as the corresponding rows in (2.2).

It is clear that the implication $HVM_{+CIM}^{+FC}\Rightarrow S$ can be reversed, whence we have the following criterion: system R is described by $HVM_{+CIM}^{+FC}$ if and only if it has a reduced coupling (4.1) subject to (4.2). For some simple systems, this has been semi-formally derived as the ‘joint distribution criterion’ by Fine [12], based on the idea of Suppes & Zanotti [16]. Note that the use of the language of random variables makes this criterion obtain essentially automatically.

For these and other simple systems (notably for the important class of the so-called *cyclic systems* [17]) other criteria have been derived, primarily in the form of inequalities involving expected values of the products of the random variables within different bunches. These additional criteria should be viewed as mere shortcuts, because in all cases when they are available and in many cases when they are not, the existence or non-existence of a reduced coupling (4.2)–(4.3) can be established directly, by means of linear programming.

This is a good place to note that some authors, having correctly observed that Bell-type inequalities require a system of jointly distributed variables, as in (4.3), and having also correctly observed that in a system of observable probabilities different bunches are not jointly distributed, have then erroneously concluded that the Bell-type theorems were wrong [18–20]. In fact, the only problem with these theorems, from the earliest ones in the 1960s all the way to the present, is that they are usually proved less than rigorously, with unacknowledged abuse of notation. When viewed as theorems about reduced couplings, their proofs are correct. The corrected proofs do not require that different bunches be jointly distributed. They only require that a system can be described by $HVM_{+CIM}^{+FC}$, the model that does preserve stochastic unrelatedness of different bunches.

## 5. Contextuality in inconsistently connected systems

CbD offers a generalised notion of (non)contextuality, one that applies to all systems of random variables, including *inconsistently connected* ones (those with disturbance, or signalling).^4^ Given a system R in (2.1), its (complete) *coupling* is defined as a random variable

5.1 $$S=\{S_{q}^{c}:c\in C,q\in Q,q≺c\},$$

such that, for every c∈C,

5.2 $$S^{c}\overset{d}{=}R^{c},$$

where

5.3 $$S^{c}:=\{S_{q}^{c}:q\in Q^{c}\}.$$

Note that calling S a random variable implies that, unlike in the system R, all components of S are jointly distributed. A system R is non-contextual if it has a coupling S in which, for every content q∈Q and any two contexts $c,c^{\prime }$ such that q≺c and $q≺c^{\prime }$, the probability

5.4 $$p[S_{q}^{c}=S_{q}^{c^{\prime }}],$$

is maximal possible. The maximum is computed for fixed distributions of $S_{q}^{c}$, $S_{q}^{c^{\prime }}$ (which coincide with the distributions of $R_{q}^{c}$, $R_{q}^{c^{\prime }}$, respectively). If such a coupling does not exist, R is contextual. If R is consistently connected, then the maximal probability for any event $S_{q}^{c}=S_{q}^{c^{\prime }}$ equals 1, and the definition reduces to the existence of the reduced coupling introduced in the previous section.

To illustrate this for our example (2.2), a coupling for $R_{0}$ is a random variable
5.5

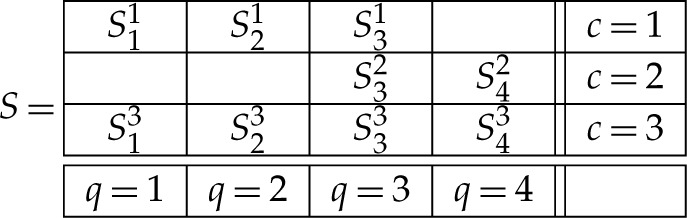

whose rows are distributed as the corresponding rows in (2.2). $R_{0}$ is non-contextual if and only if its coupling S can be chosen so that the probabilities of the events

5.6 $$S_{1}^{1}=S_{1}^{3},S_{2}^{1}=S_{2}^{3},S_{3}^{1}=S_{3}^{2},S_{3}^{1}=S_{3}^{3},S_{3}^{2}=S_{3}^{3},S_{4}^{2}=S_{4}^{3},$$

are all maximal possible. In particular, if $R_{0}$ is consistently connected, then it is non-contextual if and only if all these probabilities in some coupling S equal 1. In such a coupling, the variables $S_{1}^{1}$ and $S_{1}^{3}$ can both be renamed into $S_{1}$, the variables $S_{2}^{1}$ and $S_{2}^{3}$ can be renamed into $S_{2}$, etc. We thus obtain the reduced coupling $\left\{S_{1},S_{2},S_{3},S_{4}\right\}$ subject to (4.3).

## 6. Consistified systems

What is an HVM representation of contextuality in the case when a system may be inconsistently connected? Clearly, Bell’s $HVM_{+CIM}^{+FC}$ cannot be used, so one should choose between the two equivalent options: $HVM_{-CIM}^{+FC}$ and $HVM_{+CIM}^{-FC}$. The problem here is that these representations do not allow us to separate inconsistent connectedness from contextuality. It may seem therefore that unlike the traditional theory of contextuality, CbD cannot use HVMs as a useful descriptive tool.

However, this difficulty can be easily remedied if one replaces a system under consideration with its *consistified* equivalent [8,9]. A consistified equivalent $R^{†}$ of a system R is a consistently connected system that depicts the same empirical or theoretical situation and is contextual if and only if R is contextual. Specifically, given R in (2.1), $R^{†}$ is defined as

6.1 $$R^{†}=\{R_{\xi }^{\pi }:\pi \in C^{†},\xi \in Q^{†},\xi {≺}^{†}\pi \},$$

where

6.2 $$\begin{array}{rl} & C^{†}=\{\pi :\pi =(\cdot ,c),c\in C\}⊔\{\pi :\pi =(q,\cdot ),q\in Q\}, \end{array}$$

6.3 $$\begin{array}{rl} & Q^{†}=\{\xi :\xi =(q,c),q\in Q,c\in C,q≺c\} \end{array}$$

6.4 $$\begin{array}{rl} \mathrm{and}\; & \xi {≺}^{†}\pi ⟺\xi =(q,c)\in Q^{†}\;\&\;[\pi =(\cdot ,c)\text{ or }\pi =(q,\cdot )]. \end{array}$$

For any context π=(⋅,c), the bunch in this context is defined as

6.5 $$R^{†\pi }=R^{†(\cdot ,c)}\overset{d}{=}R^{c}.$$

To define the bunch for a context π=(q,⋅), we need an auxiliary notion. For a given q∈Q, define a random variable

6.6 $$T_{q}=\{T_{q}^{c}:c\in C,q≺c\},$$

such that for any two components $T_{q}^{c},T_{q}^{c^{\prime }}$ in $T_{q}$,

6.7 $$T_{q}^{c}\overset{d}{=}R_{q}^{c},$$

and the probability

6.8 $$p[T_{q}^{c}=T_{q}^{c^{\prime }}],$$

is maximal possible. Let us assume, for simplicity, that such $T_{q}$ exists and is unique for all q∈Q.^5^ Then, for any context π=(q,⋅), the bunch in this context is defined as

6.9 $$R^{†\pi }=R^{†(q,\cdot )}\overset{d}{=}T_{q}.$$

This completes the construction of $R^{†}$.

Clearly, a consistified system is (strongly) consistently connected: for any ξ=(q,c) it contains two distributional copies of $R_{q}^{c}$, in the contexts (⋅,c) and (q,⋅). It should also be clear, by comparing the CbD definition of (non)contextuality with the traditional definition applied to the consistified equivalent of a system, that the system and its equivalent are always contextual or non-contextual together. For a more rigorous argument, see [8].

For our example $R_{0}$ in (2.2), the consistified equivalent is (omitting commas and brackets to save space)
6.10

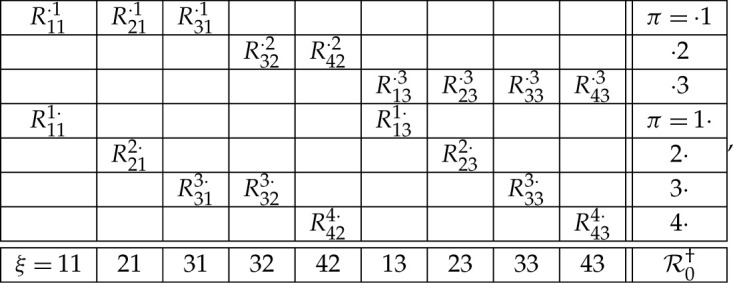

where the bunches in the first three rows are distributional copies of the corresponding rows in $R_{0}$, the distributions of the two variables in each column are identical, and in each of the last four rows the probability of the pairwise equality of its elements is maximal possible.

## 7. Equivalence theorem for consistified systems

The main reason why the notion of a consistified systems is useful is the fact that the inconsistent connectedness of a system R is eliminated in $R^{†}$ (more precisely, translated into the structure of its (q,⋅)-bunches) while its contextuality status is preserved. One can ascertain therefore whether $R^{†}$ is describable by $HVM_{+CIM}^{+FC}$ as one can with any other (strongly) consistently connected system. If it is not, then $R^{†}$ should be described by either of $HVM_{-CIM}^{+FC}$ and $HVM_{+CIM}^{-FC}$, and this time there can be no confusion as to whether they depict inconsistent connectedness or contextuality—it is definitely the latter. However, the applicability of and deviations from $HVM_{+CIM}^{+FC}$ acquire a specific form in the case of consistified systems.

It should be clear from the construction of $R^{†}$ that the indexing sets $Q^{†(\cdot ,c)}$ of different (⋅,c)-bunches are disjoint, and that the union of these indexing sets is the entire $Q^{†}$ (consisting of all ξ=(q,c) such that q≺c). This means that we can use the same function to represent all (⋅,c)-bunches,

7.1 $$R^{†(\cdot ,c)}=f(Q^{†(\cdot ,c)},X^{\left(\cdot ,c\right)}(c)),$$

where $X^{\left(\cdot ,c\right)}(c)$ for different c∈C is a set of stochastically unrelated random variables whose distributions may vary with c. By forming an arbitrary coupling X of $X^{\left(\cdot ,c\right)}(c)$ for all c∈C, we can rewrite this as

7.2 $$R^{†(\cdot ,c)}=s(Q^{†(\cdot ,c)},{Proj}_{\left(\cdot ,c\right)}X^{\left(\cdot ,c\right)})=t(Q^{†(\cdot ,c)},X^{\left(\cdot ,c\right)},(\cdot ,c)),$$

where $X^{\left(\cdot ,c\right)}$ are stochastically unrelated distributional copies of X. Since $Q^{†(\cdot ,c)}$ uniquely determines (⋅,c), the function can be rewritten as

7.3 $$R^{†(\cdot ,c)}=u(Q^{†(\cdot ,c)},X^{\left(\cdot ,c\right)}).$$

By the same argument, for all (q,⋅)-bunches we have

7.4 $$R^{†(q,\cdot )}=v(Q^{†(q,\cdot )},Y^{\left(q,\cdot \right)}).$$

The last two formulae represent the $HVM_{Gen}$ for consistified systems.

It can be easily shown that one can simplify this HVM by either making the two functions u and v one and the same function or making all $X^{\left(\cdot ,c\right)}$ and $X^{\left(q,\cdot \right)}$ variables identically distributed. For the latter option, create an arbitrary coupling Φ=(X,Y) and make its distributional copies $\Phi^{\left(\cdot ,c\right)}$ and $\Phi^{\left(q,\cdot \right)}$ for all contexts of $R^{†}$. Then

7.5 $$R^{†(\cdot ,c)}=u\left(Q^{†(\cdot ,c)},{Proj}_{1}\Phi^{\left(\cdot ,c\right)}\right)=\phi_{1}\left(Q^{†(\cdot ,c)},\Phi^{\left(\cdot ,c\right)}\right)$$

and

7.6 $$R^{†(q,\cdot )}=v\left(Q^{†(q,\cdot )},{Proj}_{2}\Phi^{\left(q,\cdot \right)}\right)=\phi_{2}\left(Q^{†(q,\cdot )},\Phi^{\left(q,\cdot \right)}\right).$$

This is the form of the $HVM_{-CIM}^{+FC}$ for consistified systems: the distribution of the hidden variables is the same for all contexts, but the observable variables depend on the type of the context, (⋅,c)-type or (q,⋅)-type. Thus, the $HVM_{-CIM}^{+FC}$ representation for $R^{†(\cdot ,2)}=(R_{32}^{\cdot 2},R_{42}^{\cdot 2})$ and $R^{†(3,\cdot )}=(R_{31}^{3\cdot },R_{32}^{3\cdot },R_{33}^{3\cdot })$ in system $R_{0}^{†}$ in (6.10) are, respectively:

7.7 $$\phi_{1}(Q^{†(\cdot ,2)},\Phi^{\left(\cdot ,2\right)})=(\phi_{1}((3,2),\Phi^{\left(\cdot ,2\right)}),\phi_{1}((4,2),\Phi^{\left(\cdot ,2\right)}))$$

and

7.8 $$\phi_{2}(Q^{†(3,\cdot )},\Phi^{\left(3,\cdot \right)})=(\phi_{2}((3,1),\Phi^{\left(3,\cdot \right)}),\phi_{2}((3,2),\Phi^{\left(3,\cdot \right)}),\phi_{2}((3,3),\Phi^{\left(3,\cdot \right)})).$$

The form of $HVM_{+CIM}^{-FC}$ for consistified system obtains by creating arbitrary couplings

7.9 $$\Psi_{1}=\{R^{†(\cdot ,c)}:c\in C\}\;\mathrm{and}\;\Psi_{2}=\{R^{†(q,\cdot )}:c\in C\},$$

and forming their distributional copies for all (⋅,c)-bunches and (q,⋅)-bunches. Note that both $\Psi_{1}$ and $\Psi_{2}$ are indexed by all $(q,c)\in Q^{†}$. Then

7.10 $$R^{†(\cdot ,c)}={Proj}_{Q^{†(\cdot ,c)}}\Psi_{1}^{\left(\cdot ,c\right)}=\psi (Q^{†(\cdot ,c)},\Psi_{1}^{\left(\cdot ,c\right)})$$

and

7.11 $$R^{†(q,\cdot )}={Proj}_{Q^{†(q,\cdot )}}\Psi_{2}^{\left(q,\cdot \right)}=\psi (Q^{†(q,\cdot )},\Psi_{2}^{\left(q,\cdot \right)}).$$

For our example with $R^{†(\cdot ,2)}=(R_{32}^{\cdot 2},R_{42}^{\cdot 2})$ and $R^{†(3,\cdot )}=(R_{31}^{3\cdot },R_{32}^{3\cdot },R_{33}^{3\cdot })$ in (6.10), the $HVM_{+CIM}^{-FC}$ representation is

7.12 $$\psi (Q^{†(\cdot ,2)},\Psi_{1}^{\left(\cdot ,2\right)})=(\psi ((3,2),\Psi_{1}^{\left(\cdot ,2\right)}),\psi ((3,4),\Psi_{1}^{\left(\cdot ,2\right)}))$$

and

7.13 $$\psi (Q^{†(3,\cdot )},\Psi_{2}^{\left(3,\cdot \right)})=(\psi ((3,1),\Psi_{2}^{\left(3,\cdot \right)}),\psi ((3,2),\Psi_{2}^{\left(3,\cdot \right)}),\psi ((3,3),\Psi_{2}^{\left(3,\cdot \right)})).$$

The falsifiable $HVM_{+CIM}^{+FC}$, describing non-contextual $R^{†}$ (hence also, non-contextual R in the CbD sense), is obtained by making $HVM_{+CIM}^{-FC}$ and $HVM_{-CIM}^{+FC}$ coincide:

7.14 $$R^{†(\cdot ,c)}=\psi (Q^{†(\cdot ,c)},\Psi^{\left(\cdot ,c\right)})$$

and

7.15 $$R^{†(q,\cdot )}=\psi (Q^{†(q,\cdot )},\Psi^{\left(q,\cdot \right)}).$$

Using again our example (6.10), the $HVM_{+CIM}^{+FC}$ representation for $R^{†(\cdot ,2)}=(R_{32}^{\cdot 2},R_{42}^{\cdot 2})$ and $R^{†(3,\cdot )}=(R_{31}^{3\cdot },R_{32}^{3\cdot },R_{33}^{3\cdot })$ is

7.16 $$\psi (Q^{†(\cdot ,2)},\Psi^{\left(\cdot ,2\right)})=(\psi ((3,2),\Psi^{\left(\cdot ,2\right)}),\psi ((3,4),\Psi^{\left(\cdot ,2\right)}))$$

and

7.17 $$\psi (Q^{†(3,\cdot )},\Psi^{\left(3,\cdot \right)})=(\psi ((3,1),\Psi^{\left(3,\cdot \right)}),\psi ((3,2),\Psi^{\left(3,\cdot \right)}),\psi ((3,3),\Psi^{\left(3,\cdot \right)})).$$

## 8. Hidden assumptions about hidden variables

The literature on HVMs and contextuality contains many attempts to explicate various assumptions underlying $HVM_{+CIM}^{+FC}$. We have seen that freedom of choice and context-independent mapping, taken separately, are not assumptions, as they are universally satisfiable. We have also seen that their conjunction is restrictive, but that it is conceptually simpler to replace it with a single assumption, one that I dubbed context-irrelevance. I will now briefly discuss two additional propositions that are sometimes presented as assumptions.

*Outcome determinism* is the assumption that hidden variables and parameters of the situation (contents and contexts) uniquely determine the observable outcomes. Some researchers find this assumption challengeable [21]. Did we not tacitly introduce this assumption somewhere in the course of the development above? The answer is no: once one consistently describes HVMs in the language of random variables, rather than events and their probabilities, outcome determinism is satisfied automatically. Unless one imposes constraints on the possible distributions of $\Lambda^{c}(c)$, either of the two unfalsifiable HVMs we have discussed, say, $HVM_{+CIM}^{-FC}$, can be constructed for any system of random variables. The very fact that the components of $R^{c}$ are jointly distributed means that there is a random variable of which all these components are measurable functions. This yields the representation (3.4).

*Factorisability* is another assumption that is often presented as central for $HVM_{+CIM}^{+FC}$ [22]. Its meaning is that, using $HVM_{-CIM}^{+FC}$ for definiteness,

8.1 $$p[\gamma (Q^{c},\Gamma^{c},c)=G\;|\;\Gamma^{c}=g]=\prod_{q\in Q^{c}}p[{Proj}_{q\in Q^{c}}\gamma (Q^{c},\Gamma^{c},c)={Proj}_{q\in Q^{c}}(G)\;|\;\Gamma^{c}=g],$$

where G is a set of values indexed by $Q^{c}$ and g is a specific value of $\Gamma^{c}$. Did we not have to use this assumption? Within our conceptual framework, we did not. Once outcome determinism is accepted as trivially satisfied, factorisability has to be accepted too. Indeed, all probabilities in this equation equal 0 or 1, and the left-hand side probability is 1 if and only if all the right-hand side probabilities are 1.

## 9. Conclusion

Let us summarise.

1. The propositions that are usually presented as the assumption of free choice and the assumption of context-independent mapping in constructing HVMs, when taken separately, are not in fact assumptions. Rather they are two inter-translatable and universally satisfiable ways of describing joint distributions of random variables in a system. Because of their equivalence and their substantive emptiness these notions are mere technical labels in HVMs: one should not take them as saying anything about freedom of choice or physical influences exerted by contexts in the sense in which these notions would be discussed in science or philosophy.
2. The conjunction of free choice and context-independent mapping is a falsifiable (and de facto inapplicable to some systems) model. However, rather than being a conjunction of two assumptions (as they were viewed, e.g. in the historic discussion [14]), it is a single assumption in precisely the same sense in which a single sentence can consist of two parts neither of which is a sentence. One can avoid using the terminology of free choice and context-independent mapping altogether, even as technical labels, by interpreting $HVM_{+CIM}^{+FC}$ as an HVM with context-irrelevance: no distributions in this model may depend on context.
3. The positions just formulated are obtained almost automatically if one systematically and carefully uses the language of random variables in discussing HVMs. This also allows one to avoid the necessity of certain additional assumptions, such as outcome determinism and factorisability. To utilise the advantages of this language one has to pay meticulous attention to the distinction between jointly distributed variables and stochastically unrelated ones. ‘Hidden variables’ are nothing more than a tool for representing jointly distributed variables as measurable functions defined on the same probability space—which is true essentially by definition. The variables from different contexts, however, cannot be presented as functions of a single source of randomness, even in the HVMs with context-irrelevance: the hidden variables in these models must still be indexed by contexts.

## Conflict of interest declaration

The author declares no competing interests.

## Data Availability

This article has no additional data.

## Footnotes

1 The notation $\Lambda^{c}(c)$ may appear excessive, but it is not. The superscript c merely indicates that the random variables in different contexts are different and stochastically unrelated (this applies to both $\Lambda^{c}$ and $R^{c}$). The superscript c therefore is universal and ineliminable. The dependence of $\Lambda^{c}$ on c as an argument means that the *distribution* of the hidden variable may be different in different contexts. This may or may not be the case in other HVMs.

2 This coupling is a random variable whose components are indexed by c and distributed as the corresponding $\Lambda^{c}$.

3 In fact, $HVM_{+CIM}^{+FC}$ predicts a more restricted form of consistent connectedness, termed in CbD *strong consistent connectedness*: for any contexts $c,c^{\prime }$ and any set of contents $I\subseteq Q^{c}\cap Q^{c^{\prime }}$, we should have identically distributed $\left\{R_{q}^{c}:q\in I\right\}$ and $\left\{R_{q}^{c^{\prime }}:q\in I\right\}$. A system that is consistently connected but not strongly so is always contextual in CbD.

4 More precisely, the current, second version of CbD, applies to arbitrary systems of *dichotomous* random variables [5,6]. However, this constraint is not relevant to the present discussion. Moreover, the discussion below could easily be generalised to a class of approaches that include CbD as a special case [8].

5 Reference [8] provides an outline of how the discussion should be modified if this is not the case. If there is more than or less than one $T_{q}$ for some of the q∈Q, one should consider a class of consistified systems $R^{†}$, each with one possible combination of the realisations of $T_{q}$. This class is deemed non-contextual if and only if at least one of its elements is non-contextual (and this happens if and only if the original system R is non-contextual in the CbD sense). In particular, if the class of $T_{q}$ is empty for some q, then the class of $R^{†}$ is empty, and it should be deemed contextual.
